# Perioperative weight gain (ΔWeight): an independent predictor of major complications after major digestive surgery

**DOI:** 10.1007/s00423-026-04054-x

**Published:** 2026-07-30

**Authors:** Maximilien Ravenel, Asaad Khadda, Emilie Zhu, Laura Didisheim, Gaëtan-Romain Joliat, Stéphanie Gonvers, David Fuks, Fabian Grass, Dieter Hahnloser, Martin Hübner, Styliani Mantziari, Emmanuel Melloul, Markus Schäfer, Hugo Teixeira, Emilie Uldry, Ismail Labgaa

**Affiliations:** 1 Faculty of Biology & Medicine (FBM), University of Lausanne (UNIL), Lausanne, Switzerland; 2 Department of Visceral Surgery, Lausanne University Hospital (CHUV), Rue du Bugnon 46, Lausanne, CH-1011 Switzerland

**Keywords:** Complications, Morbidity, Prediction, Outcomes, Biomarkers

## Abstract

**Purpose:**

Prediction of major complications (mPOC) after major digestive surgery (MDS) remains challenging. Perioperative gain of weight (ΔWeight) was identified as a promising marker of morbidity after specific types of surgery. This study aimed to assess the predictive value of ΔWeight for mPOC after MDS.

**Methods:**

Retrospective analysis of consecutive patients undergoing MDS between 2013 and 2022. MDS was defined as elective surgery with anticipated duration of ≥ 120 min. Test variable was postoperative weight change calculated on postoperative day 2, defined as ΔWeight. Complications were graded according to Clavien classification. Primary outcome was mPOC (grades > II). Secondary outcomes were overall complications, Comprehensive Complication Index (CCI) and specific subtypes of complications.

**Results:**

A total of 1402 patients were included. MPOC were reported in 392 (27.9%). Median ΔWeight was 2.0 kg [0.4–3.8]. Area under the curve (AUC) of ΔWeight for mPOC was 0.614 (*p* < 0.001) and maximal Youden index determined a cut-off of 2.6 kg, yielding a negative predictive value of 79%. Patients with ΔWeight above this cut-off showed higher rates of mPOC (38.2 vs. 20.7%, *p* < 0.001), overall complications (76.2 vs. 47.2%, *p* < 0.001), anastomotic leak (9.2 vs. 5.0%, *p* = 0.002), and higher CCI (33.7 vs. 29.6, *p* < 0.001). Multivariable analysis identified ΔWeight ≥ 2.6 kg as an independent predictor of mPOC (OR, 1.49; 95% CI, 1.03–2.16; *p* = 0.033).

**Conclusion:**

ΔWeight emerged as a risk-stratification marker across complication types and an independent predictor of major postoperative complications after MDS. This supports perioperative weight monitoring, proactive mitigation of postoperative weight gain, and future studies developing multiparametric prediction tools.

**Supplementary Information:**

The online version contains supplementary material available at https://doi.org/10.1007/s00423-026-04054-x.

## Introduction

Despite substantial progress achieved over the last decades in the perioperative management of surgical patients, major digestive surgery (MDS) remains associated with non-negligeable risk of postoperative morbidity [1–5]. Complications after surgery are of particular importance as they trigger multifaceted deleterious effects on patients, caregivers and costs [6–9]. It is therefore paramount to optimize the management of postoperative complications, from early identification to treatment. Early identification of patients at risk is a crucial step for which many research efforts have been pursued to identify new reliable markers capable to predict postoperative complications (POC). Regardless of the approaches followed by these studies (e.g. biomarkers, scores, machine learning algorithms), POC prediction remains a challenging task in which room for improvement exists [10]. Numerous studies have evaluated sophisticated tools to predict POC; however, identifying a practical, early, and cost-effective tool for routine clinical use is pivotal [11, 12]. Furthermore, predicting the most impactful adverse outcomes, such as major complications (mPOC), constitutes a key within the current body of literature. Recent data suggested that early postoperative gain of weight (ΔWeight) may be a valuable predictor of complications [13–15]. However, reports derived from small cohorts and specific types of surgery. ΔWeight presumably recapitulates the combination of a variety of pathophysiological effects like fluid shifts, excessive fluid administration, intensive inflammatory reaction and capillary leak. It displays interesting characteristics and may be used in adjunction with other markers such as C-reactive protein (CRP) [16–18] or albumin drop [11, 19–21].

This study aims to evaluate the contribution of ΔWeight to anticipate mPOC after MDS.

## Methods

### Study design

Retrospective study including all consecutive patients undergoing MDS who had provided informed consent for research in the Department of Visceral Surgery of Lausanne University Hospital (CHUV) between July 2013 and July 2022. The study protocol followed TRIPOD guidelines and was approved by the Institutional Review Board (CER-VD #2023-00073).

### Selection of patients and data collection

Inclusion criteria were age ≥ 18 years and patients undergoing MDS, defined as anticipated surgery duration ≥ 120 min, as previously described [11]. It included esophageal, gastric, liver, pancreatic and colorectal surgery. Emergency cases and patients with missing ΔWeight were excluded. Collected variables included demographics, surgical-, perioperative- and clinical details as well as laboratory tests and outcomes. Gender, documented in patient files as a binary variable (male/female), was used as a proxy for sex—recognizing that ‘gender’ reflects socially constructed identity rather than biological attributes as defined by SAGER guidelines [22]. Respiratory conditions were defined as chronic obstructive pulmonary disease, bronchiectasis or severe asthma whereas cardiovascular conditions were defined as history of acute coronary syndrome, pulmonary embolism, stroke, heart failure or arrythmia.

### ΔWeight

Patient body weight [in kilograms] was measured preoperatively (POD − 1), and every morning during the postoperative phase using chair scales; this was done and reported in patients’ chart by the nursing staff.

Postoperative weight change was identified as ΔWeight and defined as the difference between weight on POD2 and the preoperative weight; it was calculated according to the following formula [weight on POD2 – preoperative weight], as previously reported [13, 14, 23]. As weight is measured in the morning and surgical procedures typically conclude between late morning and late afternoon, the elapsed time between surgery and postoperative weight measurement is thus < 48 h. Of note, the timepoint of POD 2 was selected on purpose for the following reasons: (I) fewer missing data compared to POD 1, as weighting patients on POD 1 after MDS may be challenging or even not possible (II) likely late enough to better recapitulate fluid shifts and (III) early enough to predict mPOC instead of only demonstrating mPOC (as opposed to POD 3–4). After surgery (POD 0), patients are transferred to the standard unit or to the intermediate care unit, for the ones undergoing major procedures such as pancreatoduodenectomy, major liver resection or esophagectomy.

### Endpoints

Postoperative complications were reported until POD 30 and graded according to Clavien–Dindo classification [24]. Minor and major complications were defined as grade ≤ II and grade > II, respectively. Primary endpoint was mPOC whereas secondary endpoints included: overall complications, Comprehensive Complication Index (CCI) [25], length of stay (LoS) measured upon day of surgery [26] and specific postoperative complications (i.e. respiratory, cardiovascular, infectious, anastomotic leak and delayed return to gastrointestinal function (DRGF)). DRGF was defined as either paralytic ileus or delayed gastric emptying with the need of nasogastric tube insertion, as previously determined [27, 28].

### Statistical analysis

Continuous variables were presented as mean with standard deviation (SD) or median with interquartile range (IQR) depending on their distribution. Group comparisons for continuous variables with normal distribution were calculated using Student’s t-test or Welch’s t-test, depending on the equality of variances. The Mann–Whitney U test was used for non-normally distributed variables. Categorical variables were presented as frequencies with valid percentages and compared with the chi-square (χ²) test.

Correlations were assessed using Pearson’s (r) or Spearman’s (ρ) correlation coefficients, depending on the distribution of the variables and strength of linear association was quantified by R². Receiver operating characteristic (ROC) curve analysis was conducted to evaluate the performance of ΔWeight for major complications while the optimal cut-off was determined using the maximal Youden’s index.

Logistic regression was used to identify independent predictors of major complications. Variables with a p-value < 0.05 in univariable analysis were included in the multivariable model. Statistical significance was defined as *p* < 0.05. Hosmer–Lemeshow goodness-of-fit test was used to test model calibration. Most analyses were performed using IBM SPSS Statistics version 29.0.2.0 (IBM Corp.). R (version 2026.01.0 + 392) was used to calculate confidence intervals for sensitivity, specificity, positive predictive value, and negative predictive value and to model ΔWeight as a continuous predictor using restricted cubic splines in logistic regression.

## Results

A total of 2331 patients underwent MDS during the period of inclusion. ΔWeight was available for 1402 (60.1%) patients, that were included for final analysis. To assess potential selection bias, excluded and included groups were compared (Supplementary Table 1); both groups were comparable for a majority of variables. Differences were limited to presence of cancer prevalence, blood loss and operative duration. A detailed description of the procedures included in MDS is presented in Supplementary Table 2. Baseline characteristics of the study cohort are detailed in Table 1. Median age, BMI and surgery duration were 67.0 years [56–75], 25.1 kg/m2 [22.6–28.3] and 247 min [189–318] respectively. Women accounted for 40.1% (*n* = 562) while 462 (33%) patients had an ASA score of III-IV.

**Table 1:** Descriptive characteristics of the cohort, including median [IQR] or number (%) of: Age, Gender, Cardiovascular condition, Respiratory condition, Diabetes, Cancer, Smoking, BMI, ASA III-IV, Area of surgery (Upper GI, HPB, Colorectal), Surgical approach (Laparoscopic, Open, Conversion, Other), Surgery duration and Intraoperative blood loss

	Median [IQR] or n (%) (n = 1402)
Age (years)	67.0 [56.0-75.0]
Gender (female)	562 (40.1)
Cardiovascular condition^1^	184 (13.1)
Respiratory condition^2^	71 (5.1)
Diabetes	232 (16.5)
Cancer	1025 (73.1)
Smoking	342 (24.4)
BMI (kg/m^2^)	25.1 [22.6-28.3]
ASA score (III-IV)	462 (33.0)
Area of surgery	
- Upper-GI	- 147 (10.4)
- HPB	- 667 (47.1)
- Colorectal	- 588 (41.9)
Surgical approach	
- Laparoscopic	- 612 (43.7)
- Open	- 667 (47.6)
- Conversion	- 67 (4.8)
- Other^3^	- 56. (4.0)
Surgery duration (minutes)	247.0 [189.0-318.0]
Intraoperative blood loss (mL)	200 [70-600]

Abbreviations: American Society of Anesthesiologists; *BMI* body mass index, *GI* gastrointestinal, *HR* Hazard ratio, *IQR* interquartile range, *POD* postoperative day

Respiratory condition^1^: Chronic obstructive pulmonary disease, bronchiectasis or severe asthma

Cardiovascular condition^2^: history of acute coronary syndrome, pulmonary embolism, stroke, heart failure or arrythmia

Other^3^: Through stoma, hand assisted laparoscopy or robotic

### Perioperative change of weight (ΔWeight)

**Fig. 1:**
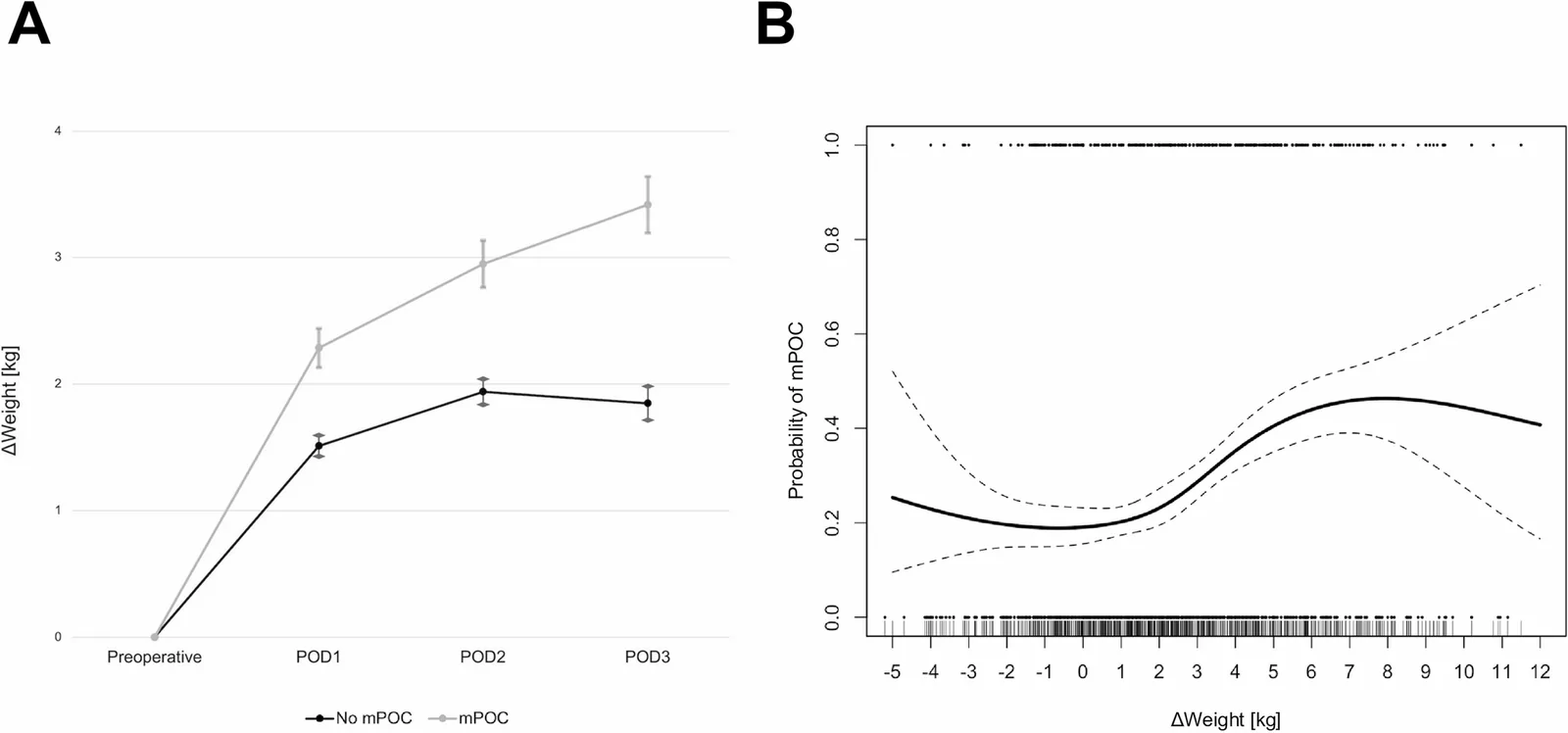
Perioperative fluctuation of weight. **A** Curves illustrate the mean values of weight fluctuation, and bars represent standard error of the mean. Black curve indicates patients without major complication and grey curve illustrates patients with mPOC. * *p*<0.001. **B** Predicted probability of major complications across ΔWeight illustrated by restricted cubic splines. Dotted lines indicate 95% confidence intervals; rug marks show the distribution of observed ΔWeight values. Abbreviations: POD: Postoperative day; mPOC: major postoperative complication

Curves illustrating the perioperative profile of weight showed an initial gain of weight on postoperative days (POD) 1 and 2 (Fig. 1A). Thereafter, the weight tended to plateau on POD 3 in patients who did not develop mPOC whereas it continued rising in patients who further developed mPOC. Moreover, patients who developed mPOC gained significantly more weight (ΔWeight) compared to those without mPOC, on POD 2 (3.5 kg [IQR: 1.1–4.8] vs. 2.0 kg [IQR: 0.3–4.5], *p* < 0.001). Median ΔWeight was 2.0 kg [IQR: 0.4–3.8] for the whole cohort. For colorectal, upper-GI and HPB surgery, median ΔWeight was 1.2 kg [IQR: -0.3-2.7], 2.0 kg [IQR: 0.8–3.5] and 2.6 kg [IQR: 1.1–4.4], respectively.

### Cut-off and predictive value of ΔWeight for major complications

The ROC curve for ΔWeight, as a continuous value, to predict mPOC yielded an area under the curve (AUC) of 0.614 [0.580–0.648] (*p* < 0.001). We then determined a cut-off value of + 2.6 kg using the maximal Youden’s Index. Application of this cut-off for mPOC yielded sensitivity, specificity, positive predictive value (PPV) and negative predictive value (NPV) of 54% (95% CI 49–59), 67% (95% CI 64–70), 38% (95% CI 34–42) and 79% (95% CI 76–82), respectively. Subgroup analysis for the different subtypes of surgery was also conducted (Supplementary Table 3). Finally, ΔWeight impact was analyzed as a continuous variable and illustrated by restricted cubic splines (Fig. 1B).

### Applications of ΔWeight to discriminate patients at risk of complications

**Fig. 2:**
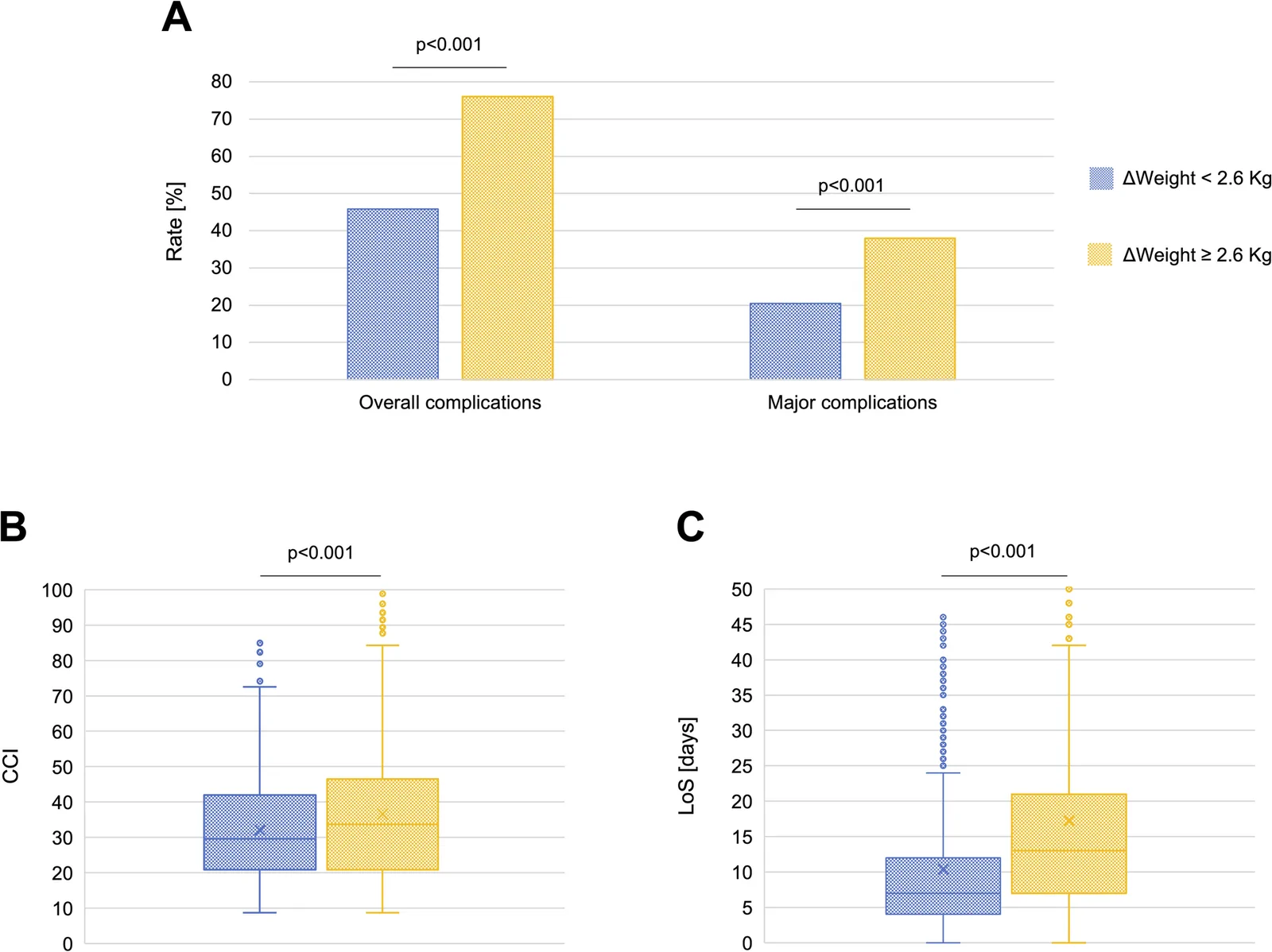
Main outcomes. Comparison of the main outcomes according to the cut-off of ΔWeight. Blue illustrates patients with ΔWeight < 2.6 kg whereas yellow illustrates patients with ΔWeight ≥ 2.6 kg. (a) Overall complications and major complications (grade ≥II according to Clavien). (b) Comprehensive complication index (CCI). (c) Length of stay (LoS)

Patients were divided and compared according to the previously established cut-off of 2.6 kg (ΔWeight < 2.6 kg vs. ΔWeight ≥ 2.6 kg) to test whether this threshold permitted to discriminate patients at higher risk of complications. Patients with ΔWeight ≥ 2.6 kg showed higher rates of overall complications (76.2 vs. 47.2%, *p* < 0.001; Fig. 2A) and mPOC (38.2 vs. 20.7%, *p* < 0.001; Fig. 2A) as well as higher CCI (33.7 vs. 29.6, *p* < 0.001; Fig. 2B) and longer LoS (14 vs. 7 days, *p* < 0.001; Fig. 2C).

**Fig. 3:**
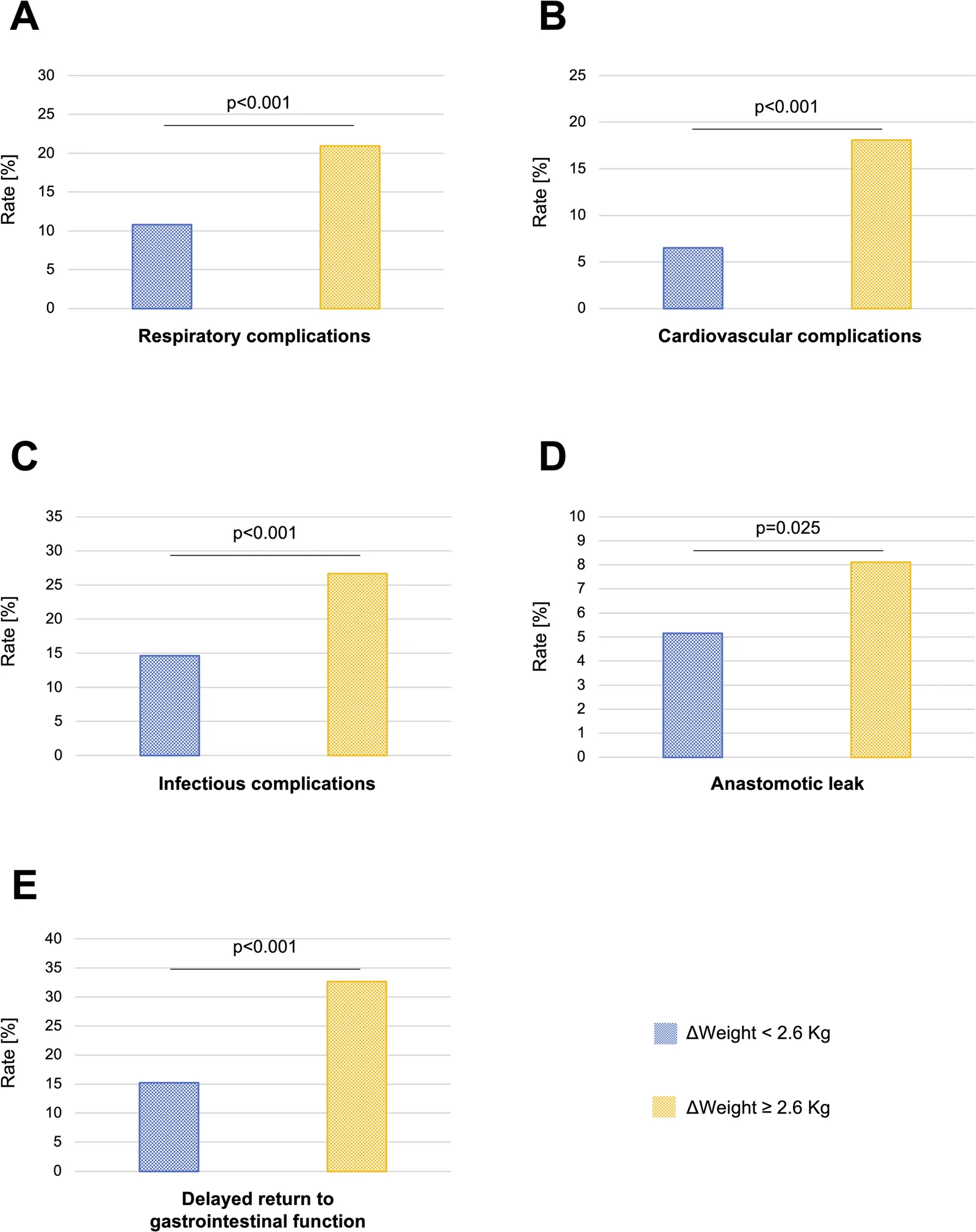
Specific subtypes of complications. Comparison of specific subtypes of complications according to the cut-off of ΔWeight. Blue illustrates patients with ΔWeight < 2.6 kg whereas yellow illustrates patients with ΔWeight ≥ 2.6 kg . (**a**) Respiratory complications. (**b**) Cardiovascular complications. (**c**) Infectious complications. (**d**) Anastomotic leak. (**e**) Delayed return to gastrointestinal function

Analysis of specific types of complications revealed consistent findings: increased rates of respiratory complication (21.4 vs. 10.6%, *p* < 0.001; Fig. 3A), cardiovascular complication (17.5 vs. 7.9%, *p* < 0.001; Fig. 3B), infection (25.6 vs. 14.8%, *p* < 0.001; Fig. 3C), anastomotic leakage (9.2 vs. 5.0%, *p* = 0.002; Fig. 3D) and DRGF (30.8 vs. 17.0%, *p* < 0.001; Fig. 3E).

### ΔWeight is an independent predictor of major postoperative complications

Uni- and multi-variable analysis were conducted to understand whether ΔWeight was a true predictor of mPOC or only a confounding factor. Analysis identified 5 independent predictive factors of mPOC (Table 2): smoking (OR 1.66; 95% CI 1.11–2.50; *p* = 0.015), Upper-GI surgery (OR 2.06; 95% CI 1.20–3.54; *p* = 0.009), surgery duration (OR 1.00; 95% CI 1.00-1.01; *p* = 0.020), CRP on POD2 (OR 1.01; 95% CI 1.00-1.01; *p* < 0.001) and ΔWeight ≥ 2.6 kg (OR 1.49; 95% CI 1.03–2.16; *p* = 0.033). The Hosmer–Lemeshow goodness-of-fit test showed a p-value of 0.537, indicating adequate calibration.

**Table 2:** Univariable and multivariable analysis of major postoperative complications. In the univariable analysis, the following parameters were analysed: Age, Gender, Cardiovascular condition, Respiratory condition, Diabetes, Cancer, Smoking, BMI, ASA III-IV, Area of surgery (Upper GI), Surgical approach (Open), Surgery duration, Intraoperative blood loss, CRP on POD 2, Total volume of intraoperative intravenous fluids and ΔWeight. Only Respiratory condition, Cancer, Smoking, BMI, ASA III-IV, Area of surgery (Upper GI), Surgical approach (Open), Surgery duration, Intraoperative blood loss, CRP on POD 2, Total volume of intraoperative intravenous fluids and ΔWeight were included in the multivariable analysis

	Univariable			Multivariable		
	OR	95% CI	*p*-value	OR	95% CI	*p*-value
Age (years)	1.01	1.00-1.01	0.066			
Gender (female)	0.83	0.66–1.06	0.141			
Cardiovascular condition^1^	1.22	0.87–1.71	0.252			
Respiratory condition^2^	2.01	1.24–3.27	0.005	1.31	0.57–3.03	0.523
Diabetes	1.09	0.80–1.49	0.560			
Cancer	1.73	1.30–2.31	< 0.001	1.41	0.87–2.15	0.179
Smoking	1.44	1.10–1.88	0.009	1.66	1.11–2.50	0.015
BMI (kg/m²)	0.97	0.95-1.00	0.020	0.97	0.93–1.01	0.130
ASA score (III/IV)	1.63	1.28–2.08	< 0.001	1.38	0.94–2.04	0.103
Area of surgery (upper GI)	3.62	2.48–5.28	< 0.001	2.06	1.20–3.54	0.009
Surgical approach (open)	1.79	1.40–2.30	< 0.001	1.33	0.81–2.18	0.267
Surgery duration (minutes)	1.01	1.00-1.01	< 0.001	1.00	1.00-1.01	0.020
Intraoperative blood loss (mL)	1.00	1.00–1.00	< 0.001	1.00	1.00–1.00	0.554
CRP on POD 2 (mg/L)	1.01	1.01–1.01	< 0.001	1.01	1.00-1.01	< 0.001
Total volume of intraoperative intravenous fluid (mL)	1.00	1.00–1.00	< 0.001	1.00	1.00–1.00	0.334
ΔWeight (≥ 2.6 kg)	2.37	1.86–3.01	< 0.001	1.49	1.03–2.16	0.033

Abbreviation: American Society of Anesthesiologists; *BMI* Body mass index, *CI* Confidence interval, *GI* Gastrointestinal, *HR* Hazard ratio, *POD* Post operative day

Respiratory condition^1^: Chronic obstructive pulmonary disease, bronchiectasis or severe asthma

Cardiovascular condition^2^: history of acute coronary syndrome, pulmonary embolism, stroke, heart failure, or arrythmia

### Determinants of ΔWeight

**Fig. 4:**
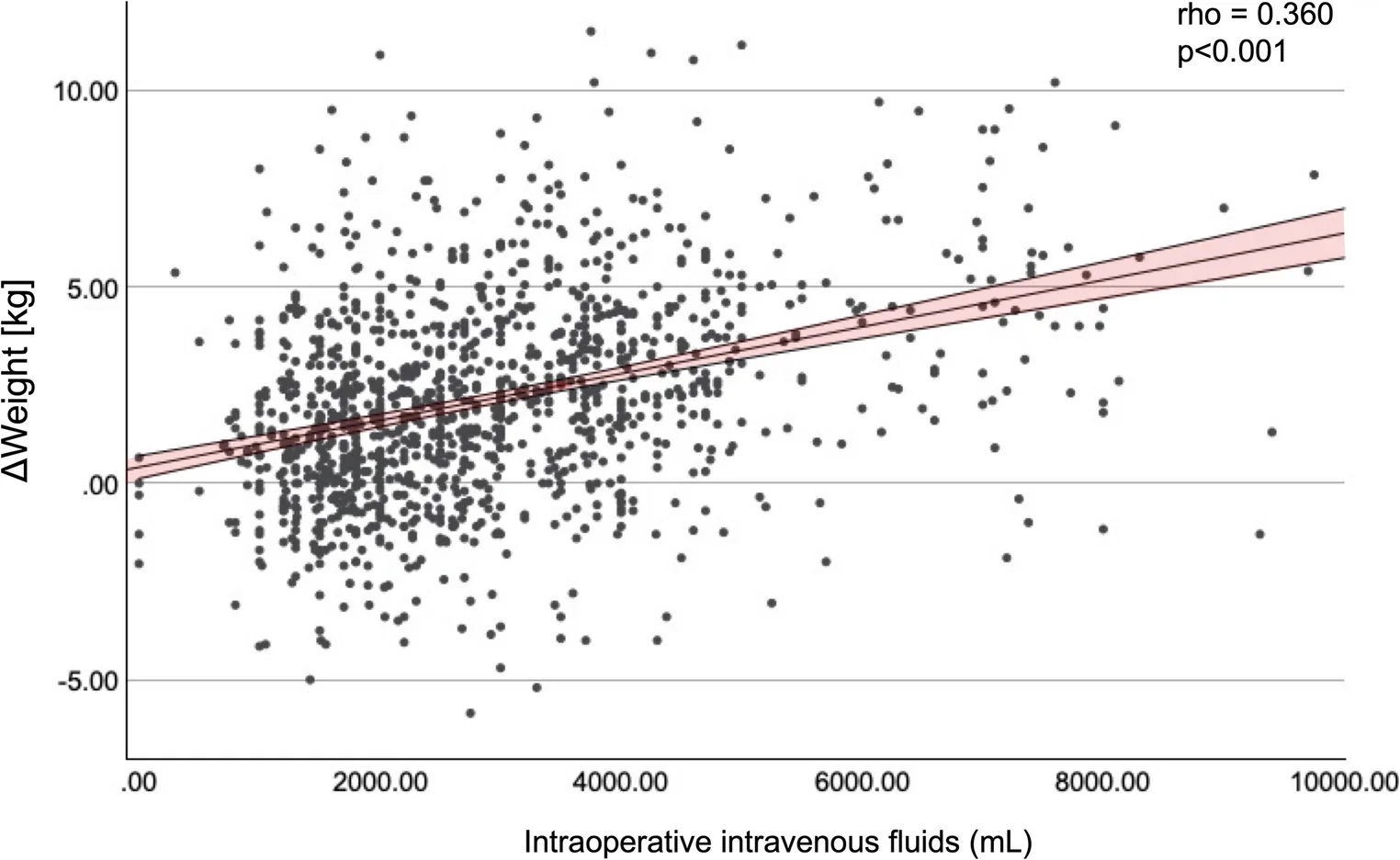
Curves illustrate the mean values of weight fluctuation, and bars represent standard error of the mean. Black curve indicates patients without major complication and grey curve illustrates patients with mPOC. * *p* < 0.001

Factors associated with a higher ΔWeight were sought. First, we tested the intuitive hypothesis that ΔWeight was intricately linked to fluid management, using correlation analysis. Results demonstrated poor correlation (rho(ρ) = 0.360, *p* < 0.001), with a corresponding R² of 0.127 (Fig. 4). A multivariable model was subsequently built to detect determinants of higher ΔWeight, identifying the following factors (Table 3): age (OR 1.02; 95% CI 1.01–1.04; *p* < 0.001), female gender (OR 1.97; 95% CI 1.5–2.59; *p* < 0.001), ASA score ≥ III (OR 1.88; 95% CI 1.40–2.51; *p* < 0.001), open surgery (OR 1.83; 95% CI 1.31–2.55; *p* < 0.001), surgery duration (OR 1.00; 95% CI 1.00–1.00; *p* = 0.034) and intraoperative volume of intravenous fluids (OR 1.00; 95% CI 1.00–1.00; *p* < 0.001).

**Table 3:** Univariable and multivariable analysis of ΔWeight ≥ 2.6 kg. In the univariable analysis, the following parameters were analysed: Age, Gender, Cardiovascular condition, Respiratory condition, Diabetes, Cancer, Smoking, BMI, ASA III-IV, Area of surgery (Upper GI), Surgical approach (Open), Surgery duration, Intraoperative blood loss, CRP on POD 2, Total volume of intraoperative intravenous fluids and Preoperative albumin. Only Age, Gender, Diabetes, Cancer, ASA III-IV, Area of surgery (Upper GI), Surgical approach (Open), Surgery duration, Intraoperative blood loss, CRP on POD 2 and Total volume of intraoperative intravenous fluids were included in the multivariable analysis

	Univariable			Multivariable		
	OR	95% CI	*p*-value	OR	95% CI	*p*-value
Age (years)	1.03	1.02–1.04	< 0.001	1.02	1.01–1.04	< 0.001
Gender (female)	1.55	1.25–1.93	< 0.001	1.97	1.50–2.59	< 0.001
Cardiovascular condition^1^	1.23	0.90–1.68	0.202			
Respiratory condition^2^	1.32	0.82–2.14	0.256			
Diabetes	1.51	1.14-2.00	0.005	1.21	0.86–1.72	0.278
Cancer	1.40	1.09–1.79	0.008	0.917	0.67–1.25	0.583
Smoking	0.87	0.67–1.13	0.293			
BMI (kg/m²)	0.98	0.96-1.00	0.051			
ASA score (III/IV)	1.97	1.57–2.47	< 0.001	1.88	1.40–2.51	< 0.001
Area of surgery (upper GI)	1.79	1.22–2.61	0.003	1.32	0.95–1.84	0.103
Surgical approach (open)	2.92	2.31–3.69	< 0.001	1.83	1.31–2.55	< 0.001
Surgery duration (minutes)	1.01	1.01–1.01	< 0.001	1.00	1.00–1.00	0.034
Intraoperative blood loss (mL)	1.00	1.00–1.00	< 0.001	1.00	1.00–1.00	0.230
Total volume of intraoperative intravenous fluid (mL)	1.00	1.00–1.00	< 0.001	1.00	1.00–1.00	< 0.001
Preoperative albumin (g/L)	1.00	0.98–1.02	0.744			

Abbreviation: American Society of Anesthesiologists; *BMI *body mass index, *CI* confidence interval, *GI* gastrointestinal, *HR* hazard ratio, *POD* post operative day

Respiratory condition^1^: Chronic obstructive pulmonary disease, bronchiectasis or severe asthma

Cardiovascular condition^2^: history of acute coronary syndrome, pulmonary embolism, stroke, heart failure or arrythmia

## Discussion

The present findings identified ΔWeight as an indicator to detect patients at higher risk of different types of complications and as an independent predictor of mPOC after MDS.

As previously demonstrated, surgical patients showed a rapid postoperative gain of weight [13, 14]. Interestingly, the available data on weight until POD 3 -conversely to our previous report on ΔWeight in kidney transplant, where we only had data until POD 2 [13] -permitted to highlight different weight profiles upon POD 2: patients who did not develop mPOC showed a plateau phase of weight after POD 2 whereas weight continued rising in the ones developing mPOC (Fig. 1). Youden index established an optimal cut-off of 2.6 kg to predict mPOC, which appeared to be lower than for liver surgery (3.5 kg) or kidney transplant (8.5 kg) [13, 14].

The use of a single threshold for MDS raises two points: first, this cut-off requires external validation before any consideration of routine clinical implementation. Second, one may argue on the use of a single cut-off, as opposed to a tailored approach using specific thresholds for each surgery subtypes. Conceptually, tailoring ΔWeight according to the surgery subtypes sounds appropriate. Of note, this approach was not followed in the present study as it was unlikely to have a major impact on performance; conversely, a single cut-off also displays advantages, including convenience in term of clinical application by digestive surgeons.

The inclusive design of the study with various types of surgery offers pros and cons. On one hand, the increased sample size with > 1400 patients was of incomparable interest with previous studies [13, 14, 29], but on the other hand, ΔWeight should conceptually be tailored to the type of surgery. As practical consequences, this cut-off of 2.6 kg may be a bit too stringent for MDS such as in HPB surgery, where median ΔWeight were higher than colorectal or upper-GI surgery, but it nonetheless demonstrated undeniable interest as a predictor of complications, even in this cohort of heterogenous surgical interventions. Concomitantly, the inclusive design eventually impacted ΔWeight performance, showing an AUC of 0.614 and a negative predictive value of 79%, as opposed to 0.74 and 94% for liver surgery [13] and 0.74 with 75% in kidney transplant [14], respectively.

The thorough analysis on the underlying mechanisms and determinants of ΔWeight permitted to add data to our understanding of this thorny question. First, it confirmed that ΔWeight poorly correlates with intraoperative intravenous fluid management (Fig. 4), as previously noticed [13, 29]. Also, the conducted multivariable analysis identified several factors independently associated with ΔWeight ≥ 2.6 kg, including intraoperative intravenous fluid (Table 3). These two points are not contradictory but rather complementary. They indicate that (I) ΔWeight is not just a proxy of fluid management, that (II) fluid management is indeed associated with ΔWeight, which altogether suggest that (III) ΔWeight results from the effects of multiple factors, including intraoperative fluid management. Furthermore, this hypothesis is in accordance with the results of the multivariable analysis for mPOC, identifying an association with intraoperative intravenous fluid on univariable analysis but not on multivariable analysis. This is of particular importance as it underpins that ΔWeight recapitulates a variety of pathophysiological mechanisms driving mPOC, such as inadequate intraoperative fluid management but also likely suboptimal nutritional status, serum albuminemia drop or abnormal capillary leak [11, 12, 19–21].

Another interesting finding was that women had a nearly 2-fold higher likelihood to show high ΔWeight, in comparison to men (Table 3). This is consistent with a study by Åkerberg et al. who explored a large dataset of the Swedish National Pancreatic and Periampullary Cancer Registry with 1174 patients undergoing pancreatoduodenectomy [30], showing that women were at higher risk to gain ≥ 2 kg on POD1, as opposed to men (*p* < 0.001); the study did not provide specific hypothesis for this finding.

The following questions naturally and synergistically arise: what is the clinical significance of these results, how ΔWeight may be integrated in clinical practice and what are the perspectives of the topic? First, ΔWeight displays valuable characteristics: (I) very easy to measure and calculate, thus reproducible, (II) early indicative, already on POD 2, (III) inexpensive, and (IV) ecological. Therefore, ΔWeight is typically a tool that may be used in any institution with surgical patients undergoing MDS, regardless of geographical location, financial and technical resources. Then, one must acknowledge that ΔWeight is not the grail and its performance was moderate. On a clinical perspective, its NPV of 79% is compelling but its AUC of 0.614 suggests that, taken alone, ΔWeight may only be of modest help. It seems that ΔWeight value foremost resides in its ability to facilitate risk stratification. With this in mind, there are different leads for future investigations: (I) to try tailoring ΔWeight and its cut-offs according to the type of surgery and the surgical approach to enhance its performance, (II) to build predictive scores that integrate ΔWeight; one could by instance consider combining ΔWeight and CRP and (III) to build decision-trees that integrate ΔWeight, along other parameters (IV) to evaluate whether ΔWeight may guide postoperative surveillance and indicate specific exams like early CT-scan and (V) to investigate measures aiming to mitigate the postoperative gain of weight (e.g. diuretics) and assess whether they can accordingly reduce the risk of mPOC.

Limitations of this study include its retrospective and monocenter design as well as the lack of validation cohort. Also, ΔWeight was only available in 60% of patients, which is relatively low. Specific reasons for missingness were sought but not identified, suggesting a random effect that likely resulted from documentation lacks.

Also, selection bias cannot be strictly excluded as patients with and without available ΔWeight showed differences in term of cancer prevalence, blood loss and surgery duration (Supplementary Table 1). Finally, intraoperative complications were not available. These events may impact mPOC and ΔWeight; it would be valuable to take them into account in future studies.

## Conclusion

In summary, this large-scale study identified ΔWeight as an independent predictor of mPOC and as an indicator of different types of complications, after MDS. Future studies are needed to determine how ΔWeight may be combined to other parameters and/or integrated in decision-making tools to offer sufficient performance.

## Supplementary Information

Below is the link to the electronic supplementary material.

Supplementary Material 1

## Data Availability

The datasets used and/or analyzed during the current study are available from the corresponding author on reasonable request.
