# Supplementary material for: Perioperative weight gain (ΔWeight): an independent predictor of major complications after major digestive surgery
Source: Langenbecks Arch Surg. 2026 Jul 30;411(1):295. doi: 10.1007/s00423-026-04054-x (PMC13713996; doi:10.1007/s00423-026-04054-x)
Supplement: Supplementary file 1 [file 423_2026_4054_MOESM1_ESM.docx]

**SUPPLEMENTARY MATERIAL**

**Title**: Perioperative Weight Gain (ΔWeight): an Independent Predictor of Major Complications After Major Digestive Surgery

**Journal:** Langenbeck’s Archives of Surgery

**Authors**: Maximilien Ravenel, M.S.^1^, Asaad Khadda, B.S.^1^, Emilie Zhu, M.D.^1, 2^, Laura Didisheim, M.D.^1, 2^, Gaëtan-Romain Joliat, M.D., Ph.D. ^1, 2^, Stéphanie Gonvers, M.S.^1, 2^, David Fuks, M.D., Ph.D.^1, 2^, Fabian Grass, M.D.^1, 2^, Dieter Hahnloser, M.D.^1, 2^, Martin Hübner, M.D.^1, 2^, Styliani Mantziari, M.D.^1, 2^, Emmanuel Melloul, M.D.^1, 2^, Markus Schäfer, M.D.^1, 2^, Hugo Teixeira, M.D.^1, 2^, Emilie Uldry, M.D.^1, 2^, Ismail Labgaa, M.D., Ph.D.^1, 2^

**Affiliations:**

1. Faculty of Biology & Medicine (FBM), University of Lausanne (UNIL), Lausanne, Switzerland.
2. Department of Visceral Surgery, Lausanne University Hospital (CHUV), Lausanne, Switzerland).

**Corresponding author**:

Ismail Labgaa, MD, PhD

Department of Visceral Surgery

Lausanne University Hospital (CHUV)

Rue du Bugnon 46

CH-1011 Lausanne

[Ismail.labgaa@chuv.ch](mailto:Ismail.labgaa@chuv.ch)

ORCID: 0000-0003-4286-2170

**Table S1: Comparisons of patients with and without available ΔWeight**

|  | **Value Median [IQR] or n (%)** | |  |
| --- | --- | --- | --- |
|  | **Available ΔWeight POD2 (n=1407)** | **Unavailable ΔWeight POD2 (n=924)** | **p-Value** |
| **Age (years)** | 67.0 [56.0-75.0] | 66.0 [54.8-74.0] | 0.184 |
| **Gender (female)** | 562 (40.1) | 360 (38.9) | 0.546 |
| **Cardiovascular condition**^1^ | 184 (13.1) | 123 (13.3) | 0.849 |
| **Respiratory condition**^2^ | 71 (5.1) | 64 (6.9) | 0.056 |
| **Diabetes** | 232 (16.5) | 132 (14.3) | 0.133 |
| **Cancer** | 1025 (73.1) | 623 (67.3) | 0.002 |
| **Smoking** | 342 (24.4) | 232 (25.1) | 0.449 |
| **Alcohol overconsumption** | 206 (14.7) | 124 (13.4) | 0.609 |
| **BMI (kg/m²)** | 25.1 [22.6-28.3] | 25.3 [22.7-28.6] | 0.656 |
| **ASA III-IV** | 462 (33.0) | 324 (35.0) | 0.293 |
| **Area of surgery**   - **Colorectal** - **Upper GI** - **Hepato-pancreatic** | - 588 (41.9) - 147 (10.4) - 667 (47.1) | - 550 (59.4) - 122 (13.2) - 254 (27.4) | <0.001 |
| **Surgical approach**   - **Laparoscopic** - **Open** - **Conversion** - **Other**^3^ | - 612 (42.7) - 667 (47.6) - 67 (4.8) - 56 (4.0) | - 398 (43.0) - 421 (45.5) - 60 (6.5) - 45 (5.0) | 0.027 |
| **Surgery duration (minutes)** | 247.0 [189.0-318.0] | 239 [175-307] | 0.010 |
| **Intraoperative blood loss (mL)** | 108 [72-177] | 200 [50-400] | <0.001 |

**Tab.S1** Comparing the patient cohorts with and without available ΔWeight from our database, using the median [IQR] or number of patients (%) for: Age, Gender, Cardiovascular condition, Respiratory condition, Diabetes, Cancer, Smoking, BMI, ASA III-IV, Area of surgery (Upper GI, HPB, Colorectal), Surgical approach (Laparoscopic, Open, Conversion, Other), Surgery duration and Intraoperative blood loss. The p-value for the comparison of both cohorts is given for every parameter

Abbreviations: American Society of Anesthesiologists; BMI: Body mass index; CCI: Comprehensive Complication Index; GI: Gastrointestinal; HR: Hazard ratio; IQR: Interquartile range; POD: Post operative day

Respiratory condition^1^: Chronic obstructive pulmonary disease, bronchiectasia or severe asthma

Cardiovascular condition^2^: history of acute coronary syndrome, pulmonary embolism, stroke, heart failure or arrythmia

Other^3^: Through stoma, hand assisted laparoscopy or robotic

**Table S2: Composition of surgical subgroups**

|  | Surgery type | Number |
| --- | --- | --- |
| **Upper-gastrointestinal** | Oesophagectomy | 88 |
|  | Gastric resections | 59 |
| **Hepatico-Pancreato-Biliary** | Minor liver resection | 198 |
|  | Major liver resection | 145 |
|  | Pancreatoduodenectomy | 222 |
|  | Distal pancreatectomy/Splenopancreatectomy | 87 |
|  | Other pancreatic surgery | 15 |
| **Colorectal** | Ileocoecal/right hemicolectomy | 89 |
|  | Left hemicolectomy/sigmoid resection | 187 |
|  | Total/Subtotal colectomy/Proctocolectomy | 66 |
|  | Anterior rectum resection | 129 |
|  | Other colorectal surgery | 117 |
| ***Total*** |  | *1402* |

**Tab.S2** Surgical procedures included in the upper gastrointestinal, hepato-pancreato-biliary, and colorectal subgroups.

**Table S3: ΔWeight predictive performance – a subgroup analysis**

|  | N | mPOC | AUC and confidence interval | p-value AUC | Cut-off value (Kg) | Maximal Youden’s Index | Se (%) | Sp (%) | PPV (%) | NPV (%) |
| --- | --- | --- | --- | --- | --- | --- | --- | --- | --- | --- |
| **UpperGI** | 147 | 73 | 0.619 [0.528-0.710] | 0.010 | 2.83 | 0.236 | 47.9 | 75.7 | 66.1 | 59.5 |
| **HBP** | 667 | 186 | 0.629 [0.581-0.677] | <0.001 | 2.89 | 0.215 | 61.8 | 59.7 | 37.2 | 80.2 |
| **Colorectal** | 588 | 126 | 0.577 [0.518-0.636] | 0.011 | 2.12 | 0.177 | 48.4 | 69.3 | 29.4 | 83.1 |
| ***MDS*** | *1402* | *385* | *0.614 [0.580-0.648]* | *<0.001* | *2.62* | *0.205* | *53.2* | *66.3* | *38.5* | *77.4* |

**Tab.S3** Subgroup analysis of ΔWeight performance for predicting mPOC across surgical specialties (Upper GI, HPB, Colorectal): AUC, sensitivity, specificity, PPV, NPV, and optimal cut-off.

Abbreviations: AUC: Area Under the Curve ; MDS: Major Digestive Surgery ; mPOC: major Post Operative Complications ; N: Number of patients ; NPV: Negative Predictive Value ; PPV: Positive Predictive Value ; Se: Sensitivity ; Sp: Specificity
